# Evaluating the additive effect of Persica and chlorhexidine mouthwashes on oral health status of children receiving chemotherapy for their hematomalignancy: A randomized clinical trial

**DOI:** 10.4317/jced.56104

**Published:** 2020-06-01

**Authors:** Zahra Bahrololoomi, Azam Sadat-Hashemi, Mohammad Hassan-Akhavan-Karbassi, Yasamin Khaksar

**Affiliations:** 1 Department of pediatric dentistry, Shahid Sadoughi University of Medical Sciences and Health Services, Yazd, Iran; 2 Department of Pediatrics, Hematology, Oncology and Genetics Research Center, Shahid Sadoughi University of Medical Sciences and Health Services, Yazd, Iran; 3 Department of Oral Medicine, Shahid Sadoughi University of Medical Sciences and Health Services, Yazd, Iran; 4 Department of pediatric dentistry, School of Dentistry, Shiraz University of Medical Sciences and Health Services, Shiraz, Iran

## Abstract

**Background:**

No definitive protocol has been introduced for treatment or prevention of chemotherapy induced mucositis. The aim of this study was to assess the additive effect of Persica and chlorhexidine on chemotherapy induced mucositis of children with hematomalignancies.

**Material and Methods:**

This randomized clinical trial was performed on 44 children aged 6 to 12 years who were under a similar maintenance chemotherapy protocol for their hematomalignancies. The clinician instructed oral hygiene cares to the patients and their parents and the severity of the mucositis and oral health status of patients were evaluated according to Oral Assessment Guide index. Then, the patients were randomly assigned to one of two experimental groups and were instructed to rinse either with Persica oral drops or normal saline, twice a day for two weeks. Subsequently, the patients in both groups were educated to rinse with chlorhexidine for 30 seconds and avoid eating for an hour. Second and third oral examinations were performed on the 8th and 15th day using the same questionnaire.

**Results:**

Comparing severity of mucositis and oral health status of patients did not show any significant difference between treatment groups in either of examination sessions (*p*>0.05). However, both treatment groups showed statistically significant oral health improvement, in terms of mucositis, plaque accumulation and gingival condition, in 14 days following mouthrinses administration (*p*<0.05).

**Conclusions:**

Both mouth-rinse combinations were effective on mucositis, plaque and gingival status of children receiving chemotherapy. However, Persica does not seem to pose additional effect on chlorhexidine in decreasing severity of chemotherapy induced mucositis.

** Key words:**Mucositis, chemotherapy, children, chlorhexidine, Persica.

## Introduction

Cancer is the third leading cause of death in Iran. Leukemia, lymphoma and CNS tumors have been shown to be the most common childhood malignancies in Iran (1,2). While cancer treatment, including chemotherapy and radiotherapy, increases survival rate of cancer patients, these modalities might potentially cause some troublesome and debilitating oral complications. Oral manifestations in children receiving chemotherapy has been reported to include mucositis and erythema, dysgeusia, gingivitis, gingival enlargement, lip cracking, dry mouth, coated tongue, infectious diseases, caries, periodontitis, cheilitis, etc (3-5).

One of the most common adverse effects of chemotherapeutic agents is mucositis (5). A wide range of incidence rate has been frequently mentioned for chemotherapy induced mucositis. However, as various chemotherapy protocols is practiced in different care centers and studies, it is not practical to predict the incidence rate of mucositis in each chemotherapy regimen. Therefore, it seems essential to determine its incidence rate in different chemotherapy protocols and each of the phase of treatment including induction, consolidation and maintenance phases.

As chemotherapeutic drugs target rapidly dividing epithelial cells, mucositis tends to be more common in pediatric population, with an incidence rate of up to 45%. Furthermore, oral mucositis has shown more rapid healing process in children in comparison to adult population (6). Oral mucositis first presents as tingling sensation and erythematous patches in the oral cavity. During the course of mucositis, these lesions might eventually develop into infectious prone ulcerations, which in neutropenic condition predispose the patient to septicemia. Mucositis might influence cancer patient’s nutritional status and quality of life and acts as a dose limiting toxicity in affected patients (7,8). Different method and therapeutic agents have been investigated for prevention and treatment of chemotherapy induced oral complications including mucositis consisting basic oral care protocol (brushing, flossing, dental visits before and during the treatment and usage of bland mouth-washes) anti-inflammatory agents, antimicrobial agents, cryotherapy, antiseptic agents, antibiotics, vitamins, cytokines, immune regulator, herbal drugs, lasers, etc (9-11). Among these, effectiveness of chlorhexidine gluconate 0.12% and 0.2%, as antimicrobial and antiplaque compounds, have been widely investigated (12-17). Chlorhexidine has been shown to be both accepTable and well-tolerated in older than 6 year old children receiving chemotherapy (18). Although no guideline has been published on the efficacy of chlorhexidine mouthwash for prevention or treatment of chemotherapy induced oral mucositis in both adults and children population receiving chemotherapy, it seems to be beneficial, as it is effective in management of gingivitis and plaque accumulation, two common oral complication in these patients due to their poor oral hygiene (9).

Recently, the trend toward rinsing with Persica oral drop (Poursina co., Iran) has been increasing, especially in Iran. Persica is a herbal oral drop containing Salvadora persica, Achillea millefolium (Yarrow) and Mentha spicatac (Mint) medicinal plants. This oral rinse can be swallowed safely and is not contraindicated during childhood and pregnancy. It has been proposed that as Salvadora persica has antibacterial, anti-inflammatory and antiulcer properties, it potentially decreases gingival inflammation and microbial count of oral cavity and results in improved oral health status. Furthermore, mint and yarrow have been shown to pose anti-inflammatory and analgesic effects (19-20).

Oral assessment guide (OAG) seems to be an appropriate scale for assessing mucositis in both adult and pediatric populations. In this regard, each of the 8 items of teeth, gingiva, buccal mucosa, lips, tongue, voice, swallow and saliva, receive score 1 (normal condition), 2 (mild changes without loss of the function or mucosal barrier) or 3 (severe changes with loss of the function or mucosal barrier). Sum of these subscales give an oral assessment score of 8 (healthy oral cavity) to 24 (21).

Due to the life threatening effects of chemotherapy induced mucositis including septicemia, it seems essential to provide an appropriate and effective oral care protocol for children under chemotherapy. Accordingly, the aim of this study was to assess the additive effect of persica on chlorhexidine mouthwash in oral health status of children receiving chemotherapy for their hematomalignancy.

## Material and Methods

-Patients:

After approval by the local Ethical Committee of Shahid Sadoughi University of Medical Sciences, a convenient sample of 44 patients referred for chemotherapy was selected. Actually, based on the similar studies and considering the significance level of 0.05 and 80% statistical power of 80%, the sample size was 20 in each group. Enrolled in this study were 6-12 year old children diagnosed with hematologic malignancies, who showed the ability to control their swallowing reflex. They should also lacked any history of asthma, allergic rhinitis and dermatitis or need for radiotherapy as part of their treatment protocol. Cases demonstrating sensitivity or infection following the application of any of the mouth-rinses or lacked sufficient cooperation in rinsing the mouth-washes were excluded from the study.

-Chemotherapy:

The antineoplastic treatment regimen of all participants was according to the BFM protocol consisting 6-Mercaptopurine, Methotroxate, Vincristine and Prednisone.

-Study design:

This was a single blinded randomized clinical trial study (IRCT2014060117935N1), in which the clinician who performed oral examination and measurements was blinded to the treatment groups. The participants could not be blinded due to the fact that persica is manufactured as oral drop and has different taste in comparison to normal saline.

After obtaining written informed consent from their parents or legal guardian, patients were randomly assigned to one of the treatment groups according to free online software http://www.randomizer.org). Oral hygiene instruction consisting tooth brushing after each meal and instructions on mouthwashes according to their corresponding group were provided by the clinician to the patients and their parents. Then, the oral condition of the patients was recorded using a questionnaire consisting OAG index. The second and third oral examinations were performed on the 8th and 15th day using the same questionnaire.

-Oral hygiene protocols.

The patients were educated to brush their teeth and rinse either with Persica oral drops (Poursina co., Iran) (10 drops in 15 ml water) or normal saline (15 ml), based on their corresponding group, twice a day (after breakfast and dinner) for two weeks. Subsequently, patients in both groups were instructed to finally rinse with 15 ml of chlorhexidine 0.2% (Shahr darou co., Iran) for 30 seconds and avoid eating and drinking for an hour.

-Statistical analysis:

Data were analyzed by means of SPSS 16 software. Demographic and clinical data were compared by the Chi-square, Wilcoxon, T-test, paired T-test and Repeated measures analysis. The criterion for statistical significance was considered as *P*<0.05.

## Results

**Figure 1:**
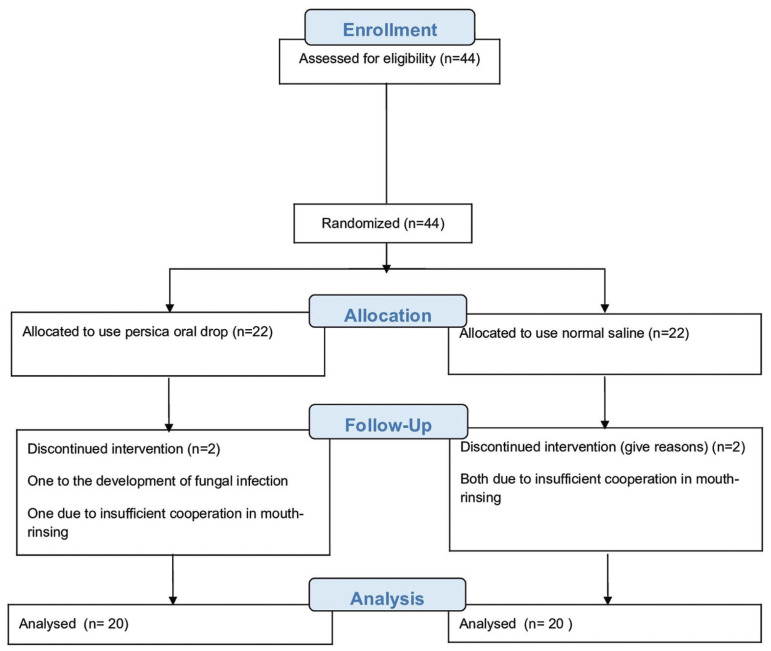
Flow diagram of the process through the phase of a randomized trial based on CONSORT statement.

Forty four children, suffering from ALL, were enrolled in this clinical trial study, 22 in each of the experimental groups. Four children were excluded from the study, 1 due to development of fungal infection and 3 due to insufficient cooperation in mouth-rinsing (Fig. 1).

According to analysis of the demographic data, no statistically significant differences were observed between the experimental groups in relation to age and gender. The mean age of participants in the group I (cholorhexidin and persica) and group II (cholorhexidin and persica) was 7.00±1.52 and 7.04±1.79, respectively. (T-test, *P*=0.451) female to male ratio of patients in the group I and II was respectively 11/9 and 7/13. (Chi-square, *P*=0.204).

**Figure 2:**
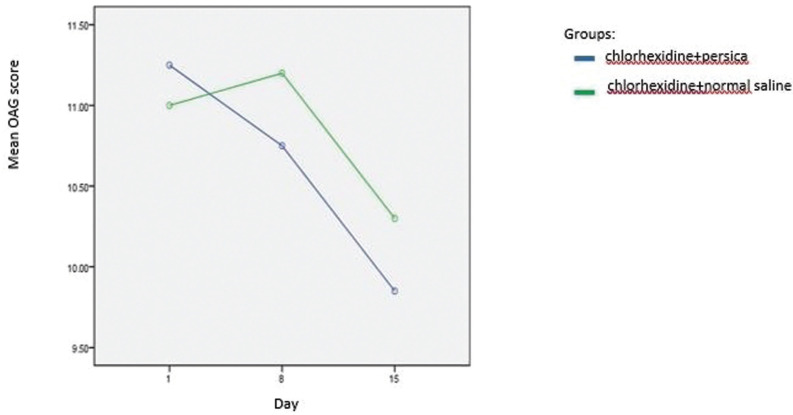
Mean oral mucositis score of the treatment groups from 1st to 15th day of investigation period.

Table 1 consists the mean OAG score of participants of each group in the 3 time points. Furthermore, the mucositis score of participants in each of the experimental groups according to OAG scale across the 3 time points is presented in Figure 2. The OAG score of the subjects tended to decrease significantly across the 3 time points.(P<0.001) However, no significant group by time interaction was observed (*P*=0.114). According to subscales of OAG score, before interventions, the prevalence of plaque and debris, gingival inflammation, mucous membranes’ erythema and redness of the tongue were respectively 80, 77.5, 15 and 7.5 percent. Furthermore, the mean value of untreated carious teeth among participants was 6.07.

**Table 1:**
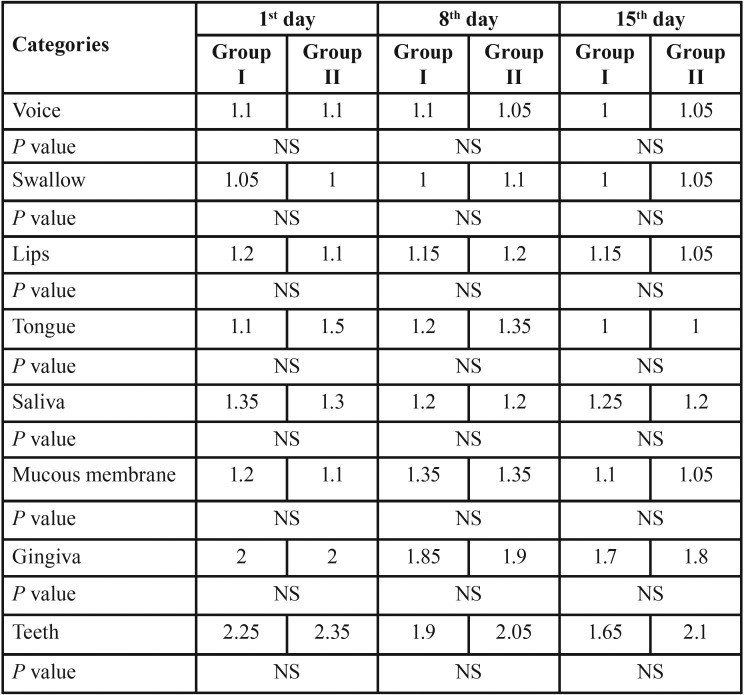
Means and *P*-values for oral assessment categories of treatment groups in evaluation sessions.

In the detailed analysis of the distribution of participants according to each of 8 factors included in OAG score (Table 2), none of them showed significant difference between treatment groups in either of sessions (*P*>0.05).

**Table 2:**
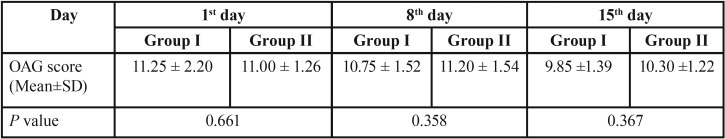
Mean values for the OAG scores in the 3 time points related to subject groups.

Gingival status of patients of both experimental groups, according to classification of OAG score, tended to improve significantly in 15 days following mouth-rinses administration. (Group I: Z=-2.449, *P*=0.014; Group II: Z=-2.000, *P*=0.046) Also as regards the status of the teeth, the plaque accumulation significantly decreased after 2 weeks rinsing with either of mouthwash combinations. (Group I: Z=-2.972, *P*=0.003; Group II: Z=-2.236, *P*=0.025).

## Discussion

Children are especially prone to develop chemotherapy induced mucositis and its occurrence has been reported to be up to 45% in pediatric population (5-6).

In the present study, the patients’ demographic data including age and sex did not show significant difference between two groups (*P*>0.05). In addition, the patients’ oral health status of the two groups had not statistically significant difference according to OAG index, which reflects patients’ oral health regarding condition of gingiva, mucous membrane and the teeth. All patients were also under the same chemotherapy regimen regarding the type and dosage of chemotherapeutic agents.

Therefore, with respect to the similarity of the samples, any kind of difference between two groups after completion of the study can be judged as the result of prescribed oral protocols.

Persica mouthwash is an inexpensive Iranian herbal product which is recently taken into consideration due to its anti-inflammatory and anti-plaque properties. It is noted that this oral drop is helpful in promoting oral hygiene and reducing plaque and gingival indices (22). Chlorhexidine gluconate has also anti- plaque, antibacterial and antifungal effects, and while coagulating serum and salivary proteins forms a white membrane on the inflamed tissues and consequently reduces the inflammation of oral mucous membranes. (16,23) Chlorhexidine is able to control gingivitis and is used frequently for removing oral plaque and bacteria (22,24). In a recent systematic review it is noted that although it is not possible to give any advice about using chlorhexidine for treatment or prevention of mucositis in the patients receiving chemotherapy, this mouthwash can be prescribed for treatment of gingivitis or helping for plaque control in these patients (9). However, in this study, prescribing chlorhexidine mouthwash instead of placebo in children under chemotherapy was based on ethical consideration as none of the children must be left without preventive protocol.

Furthermore, chlorhexidin has been frequently included in preventive protocols suggested in oral medicine literature. OAG index is the most appropriate scale for applying in clinical studies and also examining mucositis in children and its validity and reliability has been shown in children. It should be noted that the subscales which are examined in OAG index are not peculiar to mucositis, in other words this index is an appropriate criterion for assessing the patients’ oral health status (25,26).

In the present study, difference of the patients’ oral health according to OAG index following the administration of oral care and mouthwash protocols was not significant in none of the times. This shows that both protocols had similar effect on the reduction of oral mucositis in children.

It should be noted that in the present study, for homogenization of the two groups in respect of using two mouthwashes, in control group in addition to chlorhexidine, normal salinewas prescribed as negative control of Persica oral drops. It is noteworthy that normal saline is a bland and safe mouthwash that helps in the formation of granulation tissue and promotion of healing process. It is also economical, easily accessible with no serious side effects. Although, no positive impact is approved while using normal saline for controlling mucositis, this mouthwash is frequently included in prescribed protocols for promoting oral health of patients with or susceptible to mucositis and can be helpful for oral hygiene maintenance and patient comfort. In fact, patients’ oral health can be promoted by following oral care protocols and using mouthwashes for moisturizing the oral cavity, removing debris and consequently reducing accumulation of dental plaque (9). These cases can be the reason for the lack of difference between two groups in respect of children’s oral health status.

The present study is the first one that examines the additive effect of Persica on chlorhexidine in mucositis of the patients receiving chemotherapy; therefore, the results of this study are not comparable to the results of the previous studies. According to the results of this study, OAG index of the children receiving chemotherapy in both groups has been decreased significantly after two weeks of performing oral care protocol including brushing and using mouthwashes, and so it represents the promotion of oral health status of the children participating in this study. However, after one week of prescribing oral care protocol no significant change was seen in OAG index of the two groups. It should be noted that although therapeutic or preventive effect of oral care protocols and that of using chlorhexidine on mucositis of the patients receiving chemotherapy have been frequently examined (12-17), because of differences in the prescribed mouthwashes in each protocol, percent of chlorhexidine, frequency of rinsing with mouthwashes, chemotherapeutic agents, age range of the studied population, type of malignancy and small sample size in most studies, detailed comparisons between studies cannot be done.

In a study by Cheng *et al.*, oral care protocol including tooth brushing, using normal saline solution and chlorhexidine%0.2 mouthwashes, compared with control group, was more effective in preventing mucositis in children (6). In the present study, using mouthwashes containing normal saline and chlorhexidine %0.2 significantly contributed to reduction of mucositis in the population under study. However, Cheng’s study attempted to prevent mucositis in the induction phase of chemotherapy and in the present study the patients were in the maintenance phase of chemotherapy and because of the high prevalence of mucositis among patients before interventions, oral protocols were aimed to decrease the severity rather than prevention of mucositis.

In the study by Soares *et al.* in 2011, incidence rate of oral mucositis and microbial analysis of children with mucositis was examined with chlorhexidine %0.12 (17). The results of this study suggested that prophylactic use of chlorhexidine gluconate %0.12 is effective in reducing the incidence of oral mucositis and oral pathogens in children with ALL. It should be noted that in the present study, dose of chlorhexidine was %0.2 is used and due to the high prevalence of mucositis in both groups before the intervention, oral care protocol was for the treatment of mucositis.

Unlike the present study, in most studies on therapeutic or preventive effect of oral care protocols, patients’ oral health status according to OAG index during the first 5-7 days after chemotherapy had been slightly increased and then had had decreasing trend (13,27). In most of these studies, the patients receiving chemotherapy were in the induction or intensification phase of chemotherapy; therefore, due to differences in the type and dosage of the prescribed chemotherapeutic drugs, the results of these studies are different from the results of the present study.

The results of this study showed that the amount of plaque accumulation and the patients’ gingival condition according to the subscales in OAG index, were significantly improved in both groups after prescribing the mouthwashes.

The effect of chlorhexidine and Salvadora persica containing mouthwashes on the dental plaque and gingival condition of otherwise healthy patientsis examined and compared in many studies (22,28,29,30). Actually, anti-plaque and anti-inflammatory effects of Persica and chlorhexidine can be the reason for promoting the patients’ condition of teeth and gingiva and consequently promoting oral health status of the children receiving chemotherapy in the present study.

The results of this study represent high prevalence of dental caries, plaque and debris, and gingivitis in children receiving chemotherapy. Therefore, it is suggested that the children receiving chemotherapy in all phases and their parents should be aware of possible oral side effects of chemotherapy, therapeutic or preventive method for these lesions and oral health care procedures. Moreover, high prevalence of untreated dental caries in patients under study signifies the importance of the need to assess oral and dental status patients before starting chemotherapy, because chronic pulpitis and periodontitis may be the source of systemic infection during periods of myelosuppression and neutropenia.

In comparison to most of observational studies on chemotherapy induced mucositis of children, the present interventional study had an innate limitation of small number of patients appropriate for attending the study. However, hospital as a curing center for children suffering from cancer has the best potential for performing this intervention. This innate limitation of the study imposed on our will to assess greater number of patients or extending the duration of intervention or observation in future studies.

## Conclusions

Generally, the results of this study showed that both prescribed oral care protocols in children receiving chemotherapy contributes to promotion of their oral health status regarding mucositis, plaque accumulation and gingivitis. However, Persica oral drop did not show significant additive effect on chlorhexidine for reducing chemotherapy induced mucositis of children in our sample size. Therefore, to provide specific protocol for these patients, further studies are necessarily needed to overcome limitations of the present study.
